# Supplementary material for: Ranking genome-wide correlation measurements improves microarray and RNA-seq based global and targeted co-expression networks
Source: Sci Rep. 2018 Jul 18;8:10885. doi: 10.1038/s41598-018-29077-3 (PMC6052111; doi:10.1038/s41598-018-29077-3)

Ranking genome-wide correlation measurements improves microarray and RNA-seq based global and targeted co-expression networks

Supplementary Information

Franziska Liesecke[1], Dimitri Daudu[1], Rodolphe Dugé de Bernonville[1], Sébastien Besseau[1], Marc Clastre[1], Vincent Courdavault[1], Johan-Owen de Craene[1], Joel Crèche[1], Nathalie Giglioli-Guivarc'h[1], Gaëlle Glévarec[1], Olivier Pichon[1], and Thomas Dugé de Bernonville[1,*]

[1] *Université de Tours, EA2106 Biomolécules et Biotechnologies végétales, Tours, 37200, France*

[*] *corresponding author:* ***thomas.duge@univ-tours.fr***

Supplementary Table 1: Threshold values to get 10 million best gene pairs.

	PC	MI	PCC raw	SCC raw	PCC HRR	SCC HRR
microarrays	0.009373521	0.2360137	0.4781962	0.3475434	791	937
TPM	0.002825884	0.6348246	0.5097043	0.625839	1094	1305
TPM-log2	0.003280711	0.5485307	0.6999239	0.625839	1261	1305
TPM-log2-colscaled	0.003327501	5.637003	0.9197505	0.9520364	1257	1371
TPM-colscaled	0.003284566	6.015358	0.9999308	0.9999597	2054	2219
TPM-vst	0.003428413	5.643152	0.914806	0.9833948	1320	1348
counts	0.002291283	1.193767	0.7090681	0.876255	1212	1530
counts-vst	0.003402332	5.696121	0.9596237	0.9367006	1622	1847

Supplementary Table 2: Guide gene accessions. *Available as a Supplementary Dataset.*

Supplementary Table 3: Gene lists from PLC obtained with PCC-HRR Genes highlighted in yellow correspond to non-guide genes but known to be involved in the pathway. *Available as a Supplementary Dataset.*

Supplementary Table 4: Gene lists from co-occurrence networks between PCC-HRR PLC obtained with microarrays and RNA-seq TPM. Genes highlighted in yellow correspond to non-guide genes but known to be involved in the pathway. *Available as a Supplementary Dataset.*

Supplementary Table 5: Gene lists from co-occurrence networks between PCC-HRR PLC obtained with microarrays and RNA-seq TPM normalized with VST. *Available as a Supplementary Dataset.*

Supplementary Table 6: Microarray and RNA-seq accessions used in this study. *Available as a Supplementary Dataset.*

Supplementary Figure 1: Network properties in dataset-distance measurement combinations. Global network characteristics (Number of significantly enriched GO terms, global and NV AUROCs) were expressed in function of vertex or edge number. The horizontal dashed line indicates a 0.6 AUROC value taken as an arbitrary threshold separating good and poor network predictability. For each dataset, TPR=f(FPR) curves are also presented with dashed line corresponding to a random selection (with AUROC <0.5). These curves are partial and the max FPR values were obtained for 10 million gene pairs.

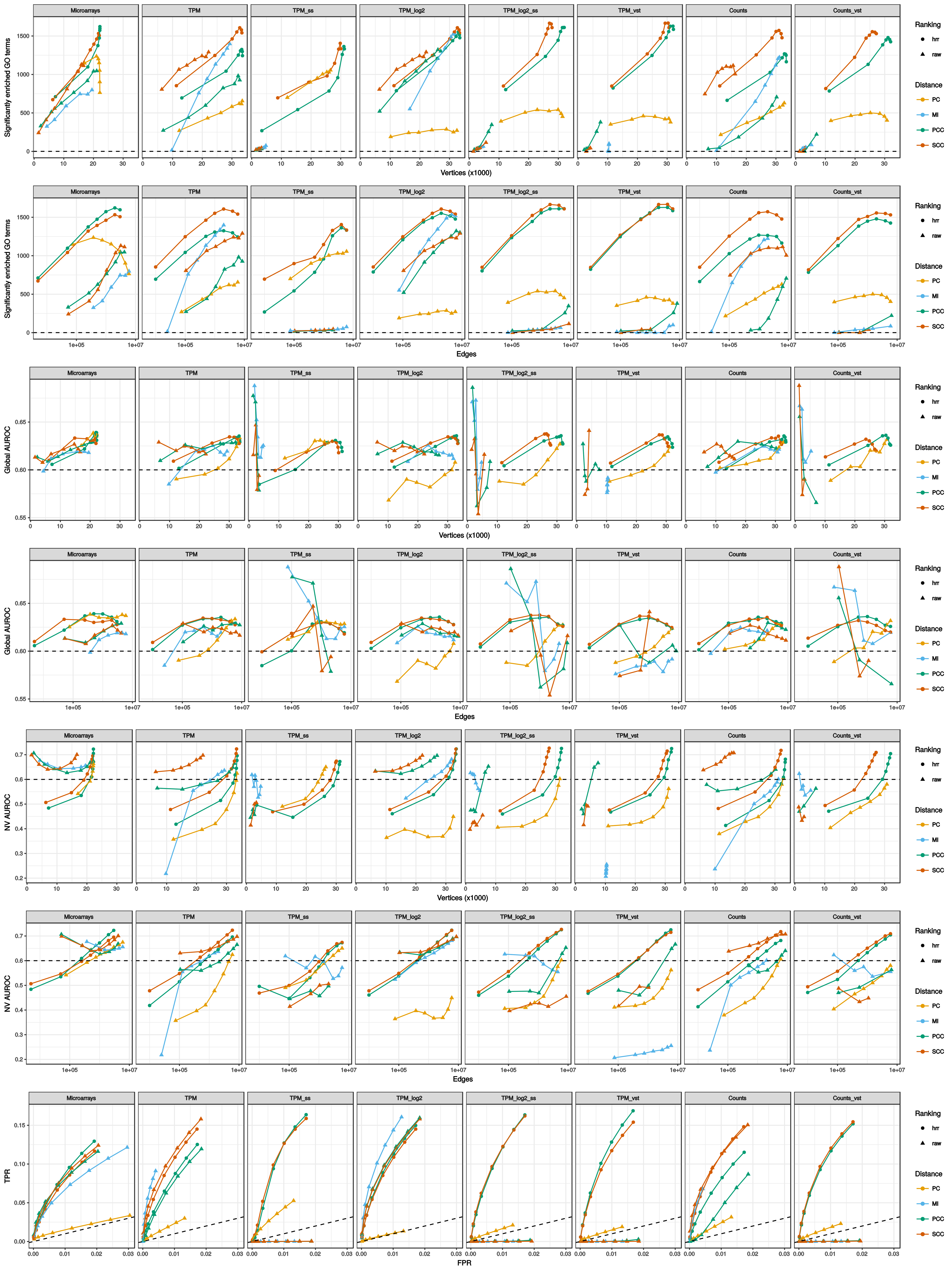

Supplementary Figure 2: Workflow for Pathway-Level Correlation. Lists of best co-expressed genes are established for each guide (or bait) gene. Redundancies among these lists (associated genes) connect guide genes to construct the PLC network. Terms 'guide gene' and 'associated genes' have been introduced by Lisso et al 2005.

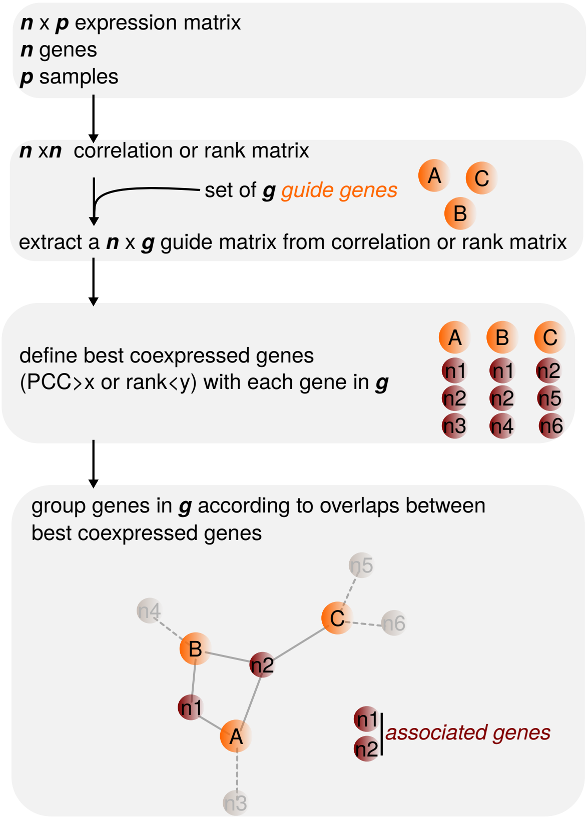

Supplementary Figure 3. PLC subgraphs for the carbohydrate (A), fatty acid (B), terpene (C) and cytokinin (D) pathways. For each PLC, the expected partitioning of guide genes is indicated in the left panel and is compared to PLC subgraphs with higher predictability and lower modularity (center; calculated with MI) or PLC subgraphs with lower predictability and higher modularity (right; calculated with PCC-HRR). Colored vertices correspond to genes encoding enzymes catalyzing steps of similar color in the expected pathway. A and B were drawn from RNA-seq TPM networks while C and D from microarray networks. Community numbers in PCC-HRR networks are indicated in deep blue and can be used to access Supplementary Table 3 online. Polygons surrounding vertices delimit communities.

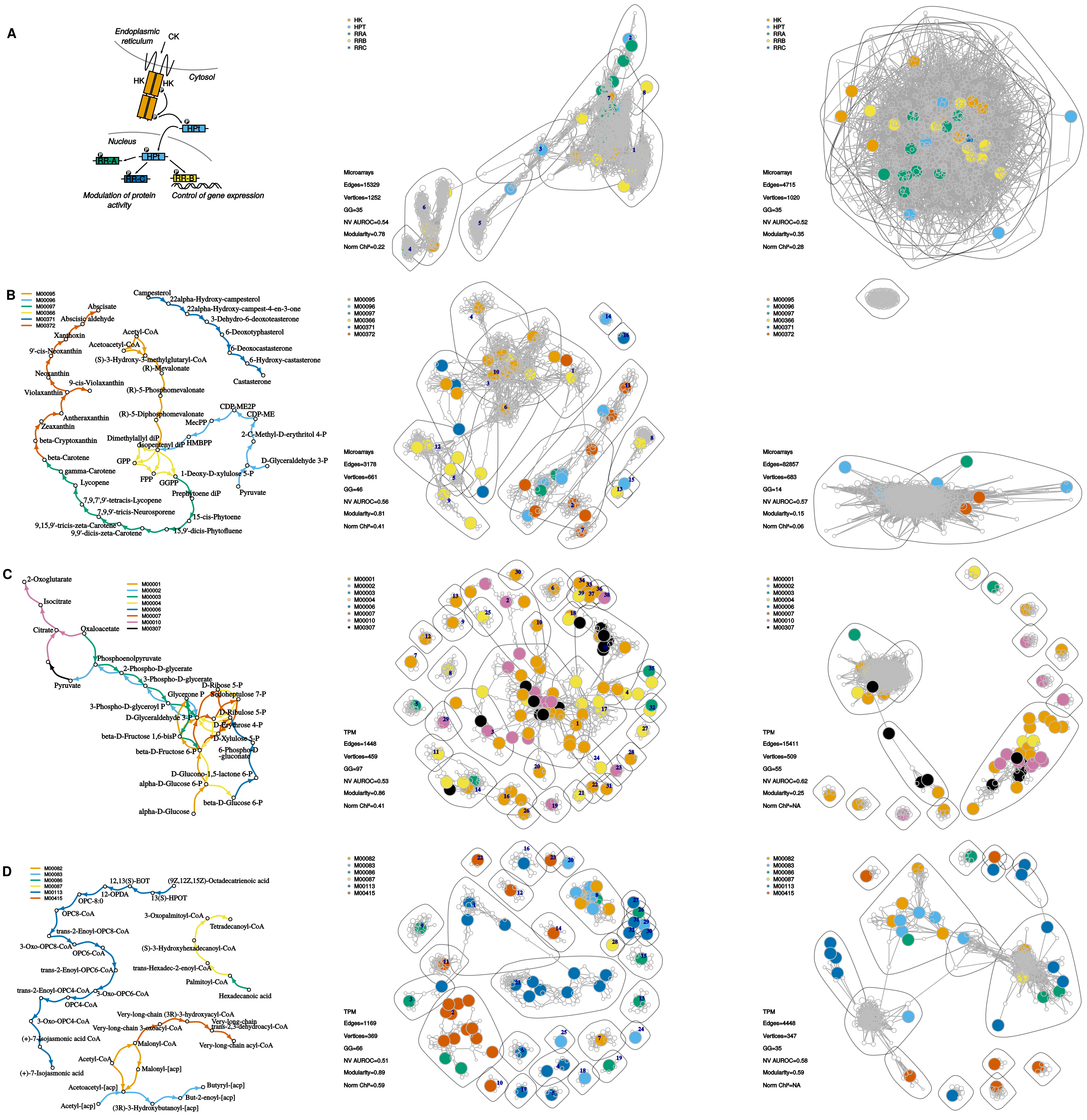

Supplementary Figure 4: PLC based on microarray and TPM data. Subgraphs were constructed with the 6 distance measurements (MI, PC, raw PCC, raw SCC, PCC-HRR and SCC-HRR) and aligned to find co-occurring edges and vertices. (A) Number of co-occurring vertices and edges. The first distance in each label correspond to microarrays and the second to TPM. Points are half-colored according to the ranking applied to the initial distance. For each intersection graph, % of guide genes (B), normalized Chi^2 statistic (agreement with expected guide gene partitioning, C), modularity (D) and NV AUROC (GO recovery performance, E) were calculated.

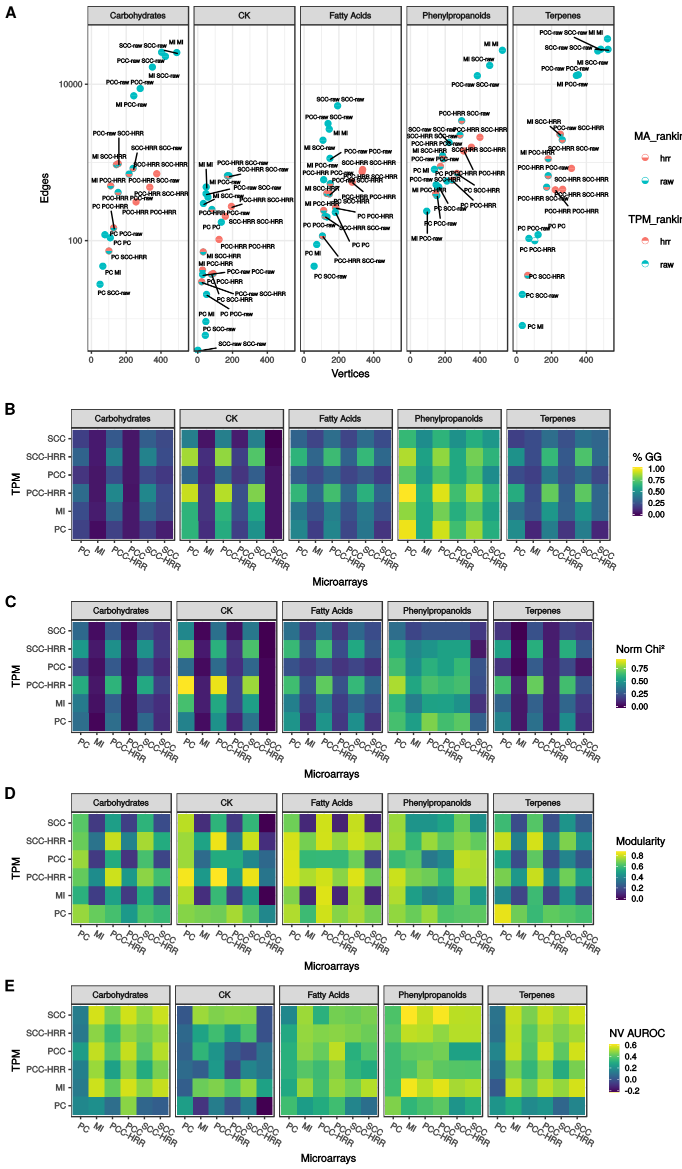

Supplementary Figure 5: Co-occurrence networks from PCC-HRR PLC constructed with microarrays and RNA-seq TPM. Community numbers are indicated in deep blue and can be used to access Supplementary Table 4 online.

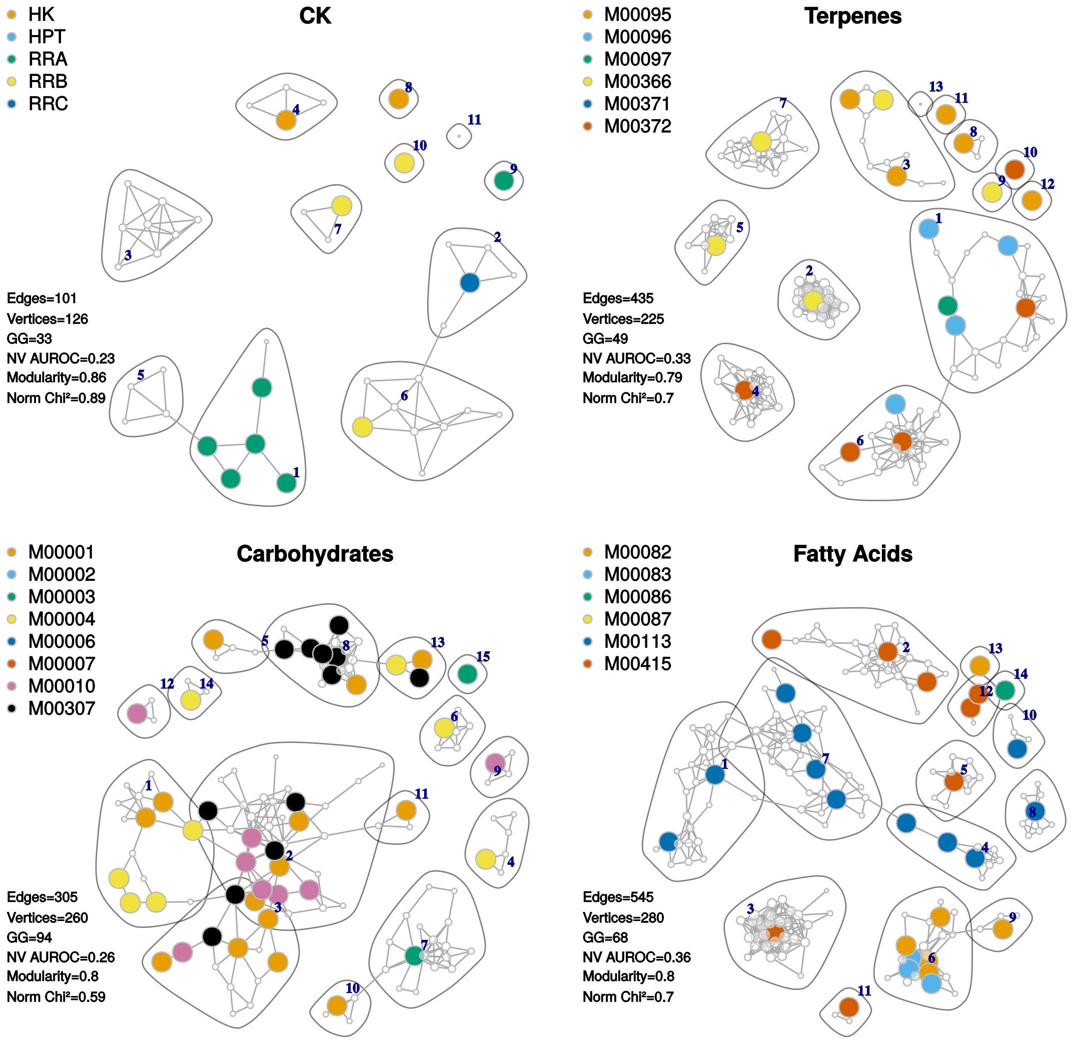

Supplementary Figure 6: Co-occurrence networks from PCC-HRR PLC constructed with microarrays and RNA-seq TPM normalized with VST. Community numbers are indicated in deep blue and can be used to access Supplementary Table 5 online.

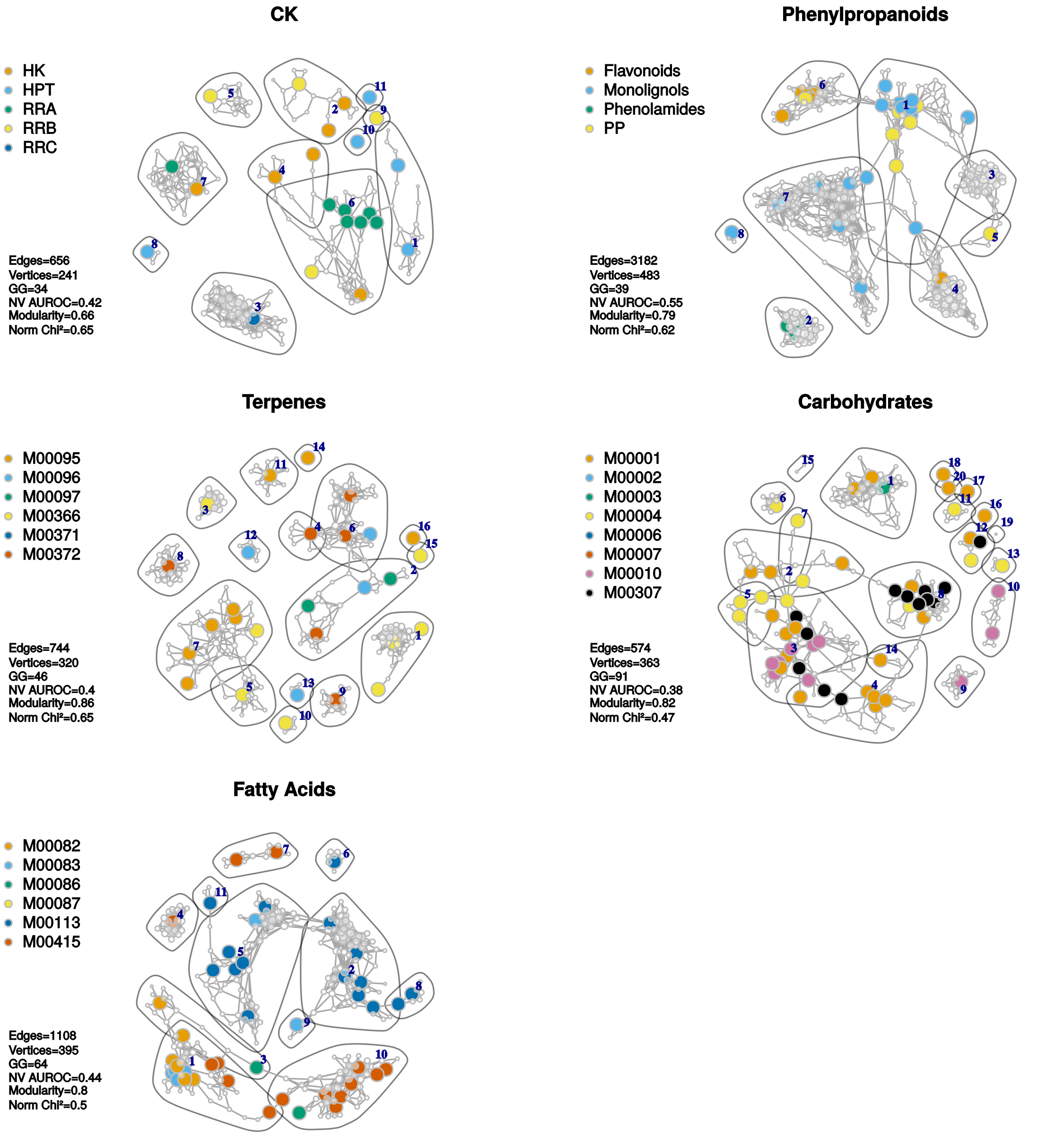

Supplement: Supplementary file 1 — Supplementary Information (contains Supplementary Table 1 & Supplementary Figures 1 to 6) [file 41598_2018_29077_MOESM1_ESM.pdf]
